# Nanotechnology-based drug delivery for the treatment of CNS disorders

**DOI:** 10.1515/tnsci-2022-0258

**Published:** 2022-12-31

**Authors:** Khushi R. Mittal, Nandini Pharasi, Bhavya Sarna, Manisha Singh, Rachana, Shazia Haider, Sachin Kumar Singh, Kamal Dua, Saurabh Kumar Jha, Abhijit Dey, Shreesh Ojha, Shalini Mani, Niraj Kumar Jha

**Affiliations:** Department of Biotechnology, Center for Emerging Diseases, Jaypee Institute of Information Technology, Noida, India; School of Pharmaceutical Sciences, Lovely Professional University, Phagwara, 144411, Punjab, India; Faculty of Health, Australian Research Centre in Complementary and Integrative Medicine, University of Technology Sydney, Ultimo, NSW, 2007, Australia; Discipline of Pharmacy, Graduate School of Health, University of Technology Sydney, NSW, 2007, Australia; Department of Life Sciences, Presidency University, Kolkata 700073, India; Department of Pharmacology and Therapeutics, College of Medicine and Health Sciences, United Arab Emirates University, Al Ain P.O. Box 15551, United Arab Emirates; Department of Biotechnology, School of Applied & Life Sciences (SALS), Uttaranchal University, Dehradun 248007, India; School of Bioengineering & Biosciences, Lovely Professional University, Phagwara, Punjab 144411, India; Department of Biotechnology, School of Engineering & Technology (SET), Sharda University, Plot No. 32-34 Knowledge Park III, Greater Noida, Uttar Pradesh, 201310, India

**Keywords:** neurodegeneration, neurodegenerative diseases, central nervous system, nanotechnology, blood–brain barrier, nanomedicine

## Abstract

Approximately 6.8 million people die annually because of problems related to the central nervous system (CNS), and out of them, approximately 1 million people are affected by neurodegenerative diseases that include Alzheimer’s disease, multiple sclerosis, epilepsy, and Parkinson’s disease. CNS problems are a primary concern because of the complexity of the brain. There are various drugs available to treat CNS disorders and overcome problems with toxicity, specificity, and delivery. Barriers like the blood–brain barrier (BBB) are a challenge, as they do not allow therapeutic drugs to cross and reach their target. Researchers have been searching for ways to allow drugs to pass through the BBB and reach the target sites. These problems highlight the need of nanotechnology to alter or manipulate various processes at the cellular level to achieve the desired attributes. Due to their nanosize, nanoparticles are able to pass through the BBB and are an effective alternative to drug administration and other approaches. Nanotechnology has the potential to improve treatment and diagnostic techniques for CNS disorders and facilitate effective drug transfer. With the aid of nanoengineering, drugs could be modified to perform functions like transference across the BBB, altering signaling pathways, targeting specific cells, effective gene transfer, and promoting regeneration and preservation of nerve cells. The involvement of a nanocarrier framework inside the delivery of several neurotherapeutic agents used in the treatment of neurological diseases is reviewed in this study.

## 1 Introduction

Brain disease difficulties involving the central nervous system (CNS) and peripheral nervous system (PNS), along with brain cancer, represent some of the most prevalent, lethal, and inadequately treated conditions. The blood–brain barrier (BBB) is interrupted in specific pathological states like strokes, Alzheimer’s disease (AD), diabetes, Parkinson’s disease (PD), seizures, and amyotrophic lateral sclerosis (ALS). Because the population of the elderly as well as adolescents with CNS problems is growing, worldwide drug development targeting brain diseases will have to rise substantially within the next 20 years [1]. When compared to all other pharmaceutical formulations, drug discovery for brain diseases has the poorest achievement. The time to develop CNS medications is substantially longer than it takes to produce non-CNS drugs.

The term “neurodegenerative disease” (NDD) is an expression used to describe a set of illnesses that mostly affect individual neurons in the neural network. The nervous system, which primarily protects the brain cord, is made up of a network of neurons, which are the basic element. Because neurons do not normally reproduce or renew spontaneously, the body is unable to repair them, and when they are damaged, they die. However, adult neurogenesis after certain forms of damage still appears to be feasible. The first proof was noted in research on rodent stress models, wherein the significantly reduced emergence of neurological precursor cells inside the adult hippocampus’s subgranular layer was imaged after sequences of highly challenging psycho-social stressors, and this reduced cell proliferation was revived by pharmacological therapy [2,3]. Antidepressants’ impact on enhanced brain progenitor cells as well as neurogenesis has also been reported in non-human primates [4]. Neurogenesis, according to Zheng et al., occurs largely in the peri-damaged regions of the brain after sustaining a traumatic brain injury in humans [5]. Such injuries are incurable, life-threatening conditions that affect nerve cell degeneration. This results in ataxias, which impacts a patient’s flexibility to shift, interact, and inhale, as well as mental functioning issues known as dementia [6]. These conditions can cause specific areas of the brain to be permanently inactive/die. In NDDs, nerve impulses and the PNS lack efficiency over a course of time but eventually die. Therapies are available, but they each have their own side effects that cannot be overcome. There are currently no documented treatments or strategies to slow the progression of neurological illnesses [7].

Clinical studies of CNS medications are challenging due to the brain’s complexity, adverse effects, and the impermeability of BBB [8]. The BBB protects the cognitive process, which is the most sensitive and intricate organ system. It protects the cerebral neurons against potentially dangerous and destructive chemicals inside the bloodstream [9] but is a substantial obstacle for medication delivery into the CNS. The BBB hinders 95% of the substances from being developed into drugs [10]. Recent research has revealed that the BBB is a variable interface that regulates the admission of chemicals from the bloodstream into the brain [11] and is a monolayer of polarized endothelial cells that are linked through complex tight junctions with astrocytes, neurons, and pericytes controlling its functioning [12]. In a healthy brain, the BBB acts as a protective covering, preventing a variety of chemicals from the bloodstream from entering into the brain, thereby ensuring proper brain function. As a result, only tiny particles can penetrate the BBB. The brain’s capillaries can be as small as 7–10 µm in diameter [13,14]. The BBB guarantees a consistent release of nutrients (including glucose, oxygen, and certain other chemicals) for brain cells and directs inflammatory cells to adapt toward changes in the surrounding environment throughout the physiologic setting. The BBB lacks intracellular and vulvar spaces, limiting the transfer of environmental information toward the cerebral [15,16,17]. Micro- and macromolecules are both being studied as therapeutic approaches for the diverse range of neurological diseases. The BBB can only be crossed by tiny molecules that have a molecular weight of <400 Da and are lipid-soluble. The overwhelming bulk of macromolecules are incapable of passing through the brain endothelium [18]. The BBB also controls the entrance of potassium (K^+^), calcium (Ca^2+^), and sodium ions (Na^+^) at synapses, maintaining minimum ocular concentrations. The BBB’s key tasks include regulating the transportation of substances from within and outside the brain, the upkeep of ionic homeostasis, and safeguarding the brain from neurotransmitter systems, systemic circulation substances, xenobiotics, and other derivatives that can affect its integrity [19,20]. The identification of a suitable nanocarrier technology is required for successful medication delivery from across BBB toward the CNS. The CNS’s appropriate operation is ensured by the tight contact involving brain microvascular endothelial cells (BMVECs) and some other constituents of the treatment system (such as neurons, astrocytes, pericytes, and basal lamina). Through physiological (tight junctions) underlying biochemical obstacles, transportation across the BBB is severely restricted (diverse transport systems and enzymes). The majority of BBB transporters are members of the ATP-binding cassette (ABC) protein superfamily, which facilitate cellular expulsion of a wide range of medicinal medicines with varying architectures and medicinal uses. P-glycoprotein (P-gp) investigations have provided us with better knowledge of the role of transporters and their contribution to the BBB [21].

The characteristics of secondary messengers dominating BBB permeability have been studied in previous studies concentrating on BBB permeability. In Ca^2+^ addition/depletion models, extracellular calcium was initially revealed to be a crucial component of tight junction (TJ) regulation [22,23]. TJ construction and operation adapt swiftly to intracellular signaling events modifying TJ complexes during physiological and pathological alterations.

In this review, we summarized different types of nanoparticles, their modes of action, and characteristics, and how they play a vital role throughout the treatment of a variety of neurological diseases. A PubMed search was performed using various keywords like central nervous system disorder, blood–brain barrier, polymeric nanoparticles, nanoparticles, solid lipid nanoparticles, liposomes, dendrimers, micelles, nanoemulsion, quantum dots, multiple sclerosis, Alzheimer’s disease, schizophrenia, Parkinson’s disease, amyotrophic lateral sclerosis, and Huntington’s disease. After this, the research papers and articles were downloaded, and information was gathered regarding the treatment for various kinds of CNS disorders and their application. The final section also covers the future aspects and recommendations.

## 2 Nanoparticles and BBB

The BBB is characterized by its own particular framework as well as the interplay of the brain’s acellular and cellular components. The BBB’s fundamental responsibility is to ensure that a suitable environment exists for the engagement as well as activation of neurons, which is essential for preserving homeostasis and modulating efflux and inflow, including shielding the brain against toxic substances [24]. The BBB comprises a persistent endothelial cell layer that is linked via gap junctions (GJs), adherent interchanges, and TJs. TJ seems to be the fundamental morphological constituent of the BBB that offers improved trans-endothelial permeability inside the BBB [25]. These junctions facilitate regulated transit of medicines across the barrier by attaching firmly to neighboring cells so that the intercellular space among them is obstructed [25,26].

Intercellular interstitial spaces appear to be almost non-existent throughout brain capillaries. Therefore, the transit likely actually occurs across all cells. As a direct consequence, lipid-soluble substances could perhaps freely permeate through all the endothelial membrane, enabling them to transfer across the BBB with ease [27]. The therapeutic benefits of encouraging prescription medication have been undermined, as cerebral abnormalities are the most resistant to treatment modalities. For the delivery of drugs throughout the BBB, lipid-soluble substances are recommended [27]. Notwithstanding a few prominent cases, just a few have advanced to the point where they can be recognized as suitable for human administration.

Approximately 95% of the pharmaceuticals evaluated fail to penetrate the BBB, yet even if they do, they do not produce any effect. This might be because the stimulants access the brain at such a slow rate that they fail to approach the therapeutic level required to have a full impact [26]. The flow of medicines including prodrugs across the BBB can also be facilitated by chemically altering compounds, modulating efflux transportation, boosting trans-cellular diffusion, and temporarily interrupting the TJ assemblies. This is implemented using techniques such as RNA interference (RNAi) to suppress protein levels [24]. These results pave the way for novel tactics or approaches in brain medication delivery.

The entry of drugs through the BBB may be through energy-dependent active targeting or via gradient-dependent passive targeting. Because of the existence of TJs linking to the adjacent cells, the water solutes passively move through them. This movement is insignificant under physiological conditions. As an alternative, trans-cellular transport could be utilized, that is, transport of the nanoparticles through the endothelial cells. The nanoparticles with a molecular weight of 500 Da are suitable for this trans-cellular transportation [28]. Drugs could be effectively and efficiently delivered to the CNS by transcytosis and by receptor-mediated delivery through the BBB. For this, a vector is required to deliver the nanoparticle in the middle of the basolateral and apical surface of the polarized cells. For the nanoparticles having size range of 100–200 nm, cargo receptor-mediated internalization, and after that, vesicular transport is an appropriate alternative. There are three steps involved in the process of transcytosis [29,30]: the first is nanoparticle endocytosis proximal to the plasma membrane. Then, intracellular vesicles transfer to the opposite surface and finally undergo exocytosis.

As discussed above, drug delivery through the BBB is not very spontaneous or favorable. Hence, there are various approaches (other than nanotechnology), which may be incorporated to enhance the rate of drug transmission to the CNS through the BBB. For instance, the use of viral vectors helps in gene transfer for gene therapy in patients suffering from neurological diseases. Viruses cannot ordinarily penetrate through BBB passively, but they can transfect their genotype into target sites. Another approach is the use of exosomes, which have a substantial advantage over all the other synthetic nanoparticles in that they are non-immunogenic, leading to a lengthy and sustainable exposure [31]. Exosomes help siRNA cross through the BBB, and yet, challenges persist for exosomes to attain their full clinical potential, along with the selection of exosome donor cells, the enhancement of the stacking protocol, the assessment of siRNA loading efficiency, toxic effects, and pharmacokinetic studies. Among these, the delivery of siRNAs to the brain stands out. Although siRNA offers immense therapeutic potential, its transport toward the brain continues to be a significant impediment. Yang et al. used a transfection reagent to separate exosomes that were recovered using brain endothelial cell growing medium when loaded with vascular endothelial growth factor (VEGF) siRNA. Furthermore, exosomes aided in siRNA crossing across the BBB as well as decreasing VEGF in xenotransplanted zebrafish with brain tumors [32].

Interestingly, the modification in the route of administration may also help in transferring drug to CNS. The intranasal technique is an efficient way to introduce medications into the brain, as the drugs rapidly pass through the olfactory pathway, bypassing the BBB [33]. According to reports, the CNS is by far the most significant HIV persistence location. To increase accessibility as well as brain absorption, Efavirenz was encapsulated inside solid nanoparticles of lipids that were homogenized under elevated heat. In comparison with oral dosing, intranasal distribution of Efavirenz nanoparticles boosted the amount of Efavirenz inside the brain by more than 150-fold [33]. Due to the obviously restricted dosage capacity through the nasal canal, the final quantity of medicine that might be administered into the brain is a constraint [34].

Recently, ultrasound has emerged as an appealing tool for facilitating medication passage over the BBB [35]. It is suggested that ultrasonic irradiations may lower the expression of TJ proteins as well as microbubbles, momentarily increasing the permeability of BBB without causing damage to brain tissues. Microbubble-enhanced diagnostic ultrasonography (MUES), a non-invasive approach, effectively assists medicines in crossing the BBB in glioma by enhancing BBB permeability [36]. MEUS elevated the development of K–Ca channels in glioblastoma, which encouraged pinocytosis and increased BBB permeability, according to Dong [37]. It allows the possibility of expanding the BBB and eliminating efflux transporters; however, the disadvantage is toxicity [36].

Nanotechnology progress may lead to increased insight into the functioning of brain pathways in addition to strategies for the evaluation and treatment of brain illnesses [38]. This seems to be particularly crucial given the inherent limitations of existing medication delivery techniques via the BBB into the CNS. Nanomaterial characteristics including decreased size, bio-compatibility, longer circulation duration, and non-toxicity have been utilized to create a revolutionary delivery mechanism capable of transporting therapeutic medicines to the brain [39,40].

Regarding specific brain locations, nanotechnology-mediated systems for drug delivery have used both selective and non-specific mechanisms [41]. Current studies have mostly concentrated on the creation of systems for the delivery of pharmaceutical substances, proteins, peptides, nucleic acids, or vaccines via nanovehicles including nanoparticles, carbon nanotubes, liposomes, micelles, or dendrimers.

The drug encapsulated in nanoparticles can sometimes not reach the BBB. To ensure that the nanoparticles reach the target site, a mediator is needed that delivers the nanoparticles to the correct location. Several studies have shown that the attachment of an antibody, surfactants, or peptide could enhance the delivery of the drug via nanoparticles through the BBB. Other ways to enhance the delivery across the BBB include the addition of polymers, proteins, glycoproteins like lactoferrin, and integrin-binding peptide or cell-penetrating peptide (CPP) [42]. Once these agents are conjugated with the nanoparticles, they recognize and bind to the specific area where the drug is needed. Gao et al. demonstrated the delivery of drug using PEG-(poly(ε-caprolactone)) nanoparticles that contained docetaxel. The nanoparticle was conjugated with TGN peptide (seq: TGNYKALHPHNG) and AS1411 aptamer, which was found to specifically target BBB ligands. Increased permeability in the case of a TGN-peptide-displaying phage was observed, and AS1411 was found to accumulate in the brain [43]. Prades et al. discovered two shuttle peptides, GPWVPSWMPPRHT and PWVPSWMPPRHT, that were composed of d-amino acids. These peptides were non-toxic, biodegradable, and able to pass through the BBB, thereby proving their efficiency in drug delivery [44]. Oller-Salvia et al. found that venom-derived cyclic peptides could be used as conjugates. This study showed that monocyclic lactam-bridged peptidomimetic MiniAp-4 derived from Apamin, that is, a bee venom-derived neurotoxin, could be used as a conjugate with gold nanoparticles and deliver the drug across the BBB [45]. Johnsen et al. reported the use of transferrin receptor-targeted gold nanoparticles (TfR-targeted AuNPs) for treating neurological diseases [46]. The uptake of TfR-targeted AuNPs was remarkably enhanced when conjugated with an antibody. They concluded that the use of a monovalent ligand could help in delivering nanoparticles for the treatment of brain diseases. The increase in nanoparticle uptake efficiency was also measured in one more study. An investigation was conducted to observe the transport and uptake AuNPs and cargo-loaded liposomes. An increase in the uptake of nanoparticles with the highest density ligand was observed, and this nanoparticle was able to pass through the BBB. The experiment was conducted *in vivo* and *in vitro* using the murine BBB [47].

## 3 Types of nanoparticles delivering drugs to CNS

Nanotechnology is an effective solution for delivering drugs to treat CNS disorders. Due to their nanosize, these engineered biodegradable and biocompatible nanoparticles can pass the BBB, and in order to achieve maximum compatibility between drug-to-be-loaded and nanoparticles, the surface of nanoparticles can also be easily modified [48]. The nanoparticle consists of two parts: One is responsible for the encapsulation of drug guarding against the enzymatic degradation, targeting the particular cells of the brain, passaging through the BBB, and releasing the drug at a specific pH. The other part is the nano-engineered complex. Nanoparticles possess a site-specific drug delivery mechanism to cross the BBB, which is one of their advantages. In a study on rats, nanoparticles of poly(butyl cyanoacrylate) were found to suppress the phenytoin resistance that is associated with P-gp [49]. There are different kinds of nanoparticles, with different functions and compositions, such as dendrimers, liposomes, micelles, solid–lipid nanoparticles (SLNs), and PNPs [50,51,52].

### 3.1 Liposomes

Liposomes are small first-generation colloidal spherical vesicles. These are composed of hydrophilic components, and at the center, there are one or more lipid bilayers, which is why they resemble the cell membrane morphologically and can be used for drug delivery [52,53]. Depending upon the number of bilayers and size, they are generally grouped into three categories: small unilamellar that ranges between 10 and 50 nm, large unilamellar that ranges between 50 and 1000 nm, and multilamellar that ranges between 20 and 100 nm [54].

Liposomes can be applied in targeting brain cancers since they possess the ability to pass across the BBB and deliver a sufficient amount of drug to the target site. Several studies have reported the use of liposomes to deliver anti-cancer drugs like methotrexate [55], 5-fluorouracil [56], paclitaxel [57], doxorubicin [58,59], and erlotinib [58]. To improve the liposome’s efficiency to pass through the BBB, several approaches could be applied. When liposomes were coated with several molecules like poly(ethylene glycol), the circulation time in the BBB passage was observed to increase [60]. Furthermore, when transferrin was used as the targeting receptor, the carrier’s translocation across the BBB was enhanced [56,58], and when glucose–vitamin C complex was incorporated, liposome accumulation at the target site increased [57].

### 3.2 PNPs

PNPs are colloidal polymer mixtures that are biocompatible and biodegradable. The core is composed of a dense polymer matrix that encloses lipophilic drug, which provides steric stabilization to the nanoparticles. The drug can be engrossed, encapsulated, or linked to the surface chemically for delivery [59]. Poly(allylamine) hydrochloride, lactide-*co*-glycolic, chitosan, poly(ethylene imine), etc. are some of the polymers commonly used for nanoparticle synthesis for brain delivery. An *in vitro* study showed how PNPs could be used for delivering of curcumin to treat AD and enhance the delivery of the drug to the brain along with reducing inflammation, plaque formation, and oxidative stress [61]. Another study showed their cytotoxic effects on cancerous cells by allowing efficient internalization of doxorubicin in human glioma cells [62].

### 3.3 SLNs

SLNs are aqueous colloidal mixtures comprising lipids such as triglycerides and fatty acids. When these nanoparticles are disseminated in solution, they gain the ability to turn into solid form while cooling [24,63]. SLNs are being assessed for drug delivery to the brain as they are physically stable, protect the drugs from being altered, and have low cytotoxicity.

SLNs can encapsulate both hydrophilic and lipophilic drugs, and the encapsulated drugs are more stable within SLN in comparison with the PNPs; therefore, a prolonged release profile of months to years is possible. Compared with PNPs, SLNs prohibit leakage and protect the active drug component from degradation (photochemical, oxidative, and chemical degradation) by immobilizing it within, resulting in a stable formulation [64]. The bioavailability of hydrophobic drugs is reported to be improved when they are in the formulation of SLNs in comparison with PNP encapsulation due to the physiological stability of the lipids [65].

### 3.4 Micelles

Micelle size ranges from 5 to 50 nm. They are amphiphilic molecules and can be non-polymeric or polymeric in nature. Polymeric micelles are composed of amphiphilic polymers and are more stable than non-polymeric micelles [52]. The structure of micelles is a core–shell type that consists of an outer hydrophobic core and an inner hydrophobic core. The outer core is composed of polyethylene glycol (PEG), whereas the inner core is composed of molecules of polypropylene glycol, polycaprolactone, and fatty acids as they permit the packaging of hydrophobic drugs [66]. The formation of micelles depends on the solution temperature and concentration [67]. Micelles have been identified as a good way to deliver poorly water-soluble molecules, since they provide controlled and sustained release, improved drug bioavailability, and maintain physical and chemical stability of the drug [68]. Micelles possess the ability to enhance drug delivery through the BBB for brain therapy. Numerous block copolymers like poly(ethylene glycol)-*b*-poly(lactic acid), distearoyl-sn-glycero-3-phosphoethanolamine-*N*-methoxy poly(ethylene glycol), and poly(styrene)-poly(acrylic acid) have been found. These copolymers showed enhancements in uptake, which proved the potential of the polymeric micelles for delivering drugs to the target site [69].

### 3.5 Nanoemulsion (NE)

NEs are a colloidal system that generally consists of edible/cooking oils, surfactant, and water. Their range varies from 100 to 500 nm [70]. They are promoted to be used as drug delivery systems to tackle several problems like inadequate identifiability and ability to penetrate the BBB [71]. NEs are less soluble in water and less stable at varied pH, temperature, and salinity. They also have low onset of action. Due to this reason, NEs are preferred for nasal delivery of the drugs.

### 3.6 Dendrimers

Dendrimers are well known for their organized shape and adaptability in delivering drugs. These are emerging polymeric structures and have various branches, called “generations.” Dendrimers have been advanced to a vast extent on the basis of branches, complexity, and component materials, in which dendrimers like polypropylene imine, polyamidoamine (PAMAM), and poly-l-lysine are highly in demand for hydrophilic and hydrophobic drug delivery. These dendrimers may show toxicity due to their cationically charged surface with negatively charged biological membranes.

PAMAM dendrimers are favored for treating brain diseases. An anti-epileptic drug named carbamazepine has been reported to be used for treating AD [72]. PAMAM dendrimers conjugated with PEG have also been reported as a way to deliver drug and reduce clotting of blood in the treatment of ischemic stroke [73]. In a study, high dendrimer accumulation, which is associated with the high level of proinflammatory cytokines, of generation 6 (G6) hydroxy PAMAM dendrimers possessing properties like targeting of injured neuronal cells and microglia was used for delivering drugs across the BBB. For cerebral palsy treatment, the inhibition of neuroinflammation mechanism was studied, with the severity of the disease, and dendrimer uptake was correlated [74]. Capmul is an enhancer that could be used to enhance the absorption to the brain and could be used for the development of *N*-acetyl-l-cysteine-conjugated dendrimers for treating neuroinflammation [75].

### 3.7 Quantum dots

Quantum dots (QDs) are semiconductors, which are of the size of nanoparticles. They have superfine sizes with diameters ranging from 2 to 10 nm. They have different electronic and optical properties like high emission, photostability, high quantum yield, size-tunable, and emission of bright light and fluorescence. It is because of these properties that QDs are considered best for treating CNS disorders. There are some conjugated QDs that are used for the diagonals and imaging of brain disorders. The chemo-electric and size of these semiconductors do not affect the BBB. The main factor allowing the transfer of QDs through the BBB is their surface. In their research, Lien et al. found that graphene QDs can stop Aβ1-42 peptides’ build-up and presented several different therapeutic applications for this problem.

There are three types of QDs:

1. Inorganic QDs: They have a continuous absorption spectrum;
2. Carbon-based QDs: They have high water solubility, biocompatibility, catalytic properties, and superficial chemical modifications;
3. Perovskite QDs: They are semiconductor materials and also have luminescent efficacy.

QDs can be made to pass through the BBB in numerous ways, and to increase their barrier-crossing ability, they are required to be associated with some molecules. However, there are some exceptions such as carbon dots (CDs). CDs are <10 nm in size and have surface passivation. For the synthesis of these dots, pyrolysis of d-glucose and l-aspartic is required. This pyrolysis significantly alters the CDs, allowing them to affect C6 glioma cells within brains. Because of the different properties like biocompatibility, robust adherent fluorescence, and simple synthesis procedure, CDs have promising potential in medicines [76,77,78,79,80,81]. Core–shell nanoparticles like Fe_2_O_3_@CDs have characteristics like good dispersity and high surface area, and are widely used for drug delivery. They are made up of a core and a shell [82,83,84].

## 4 Mode of action of nanoparticles

**Figure 1:**
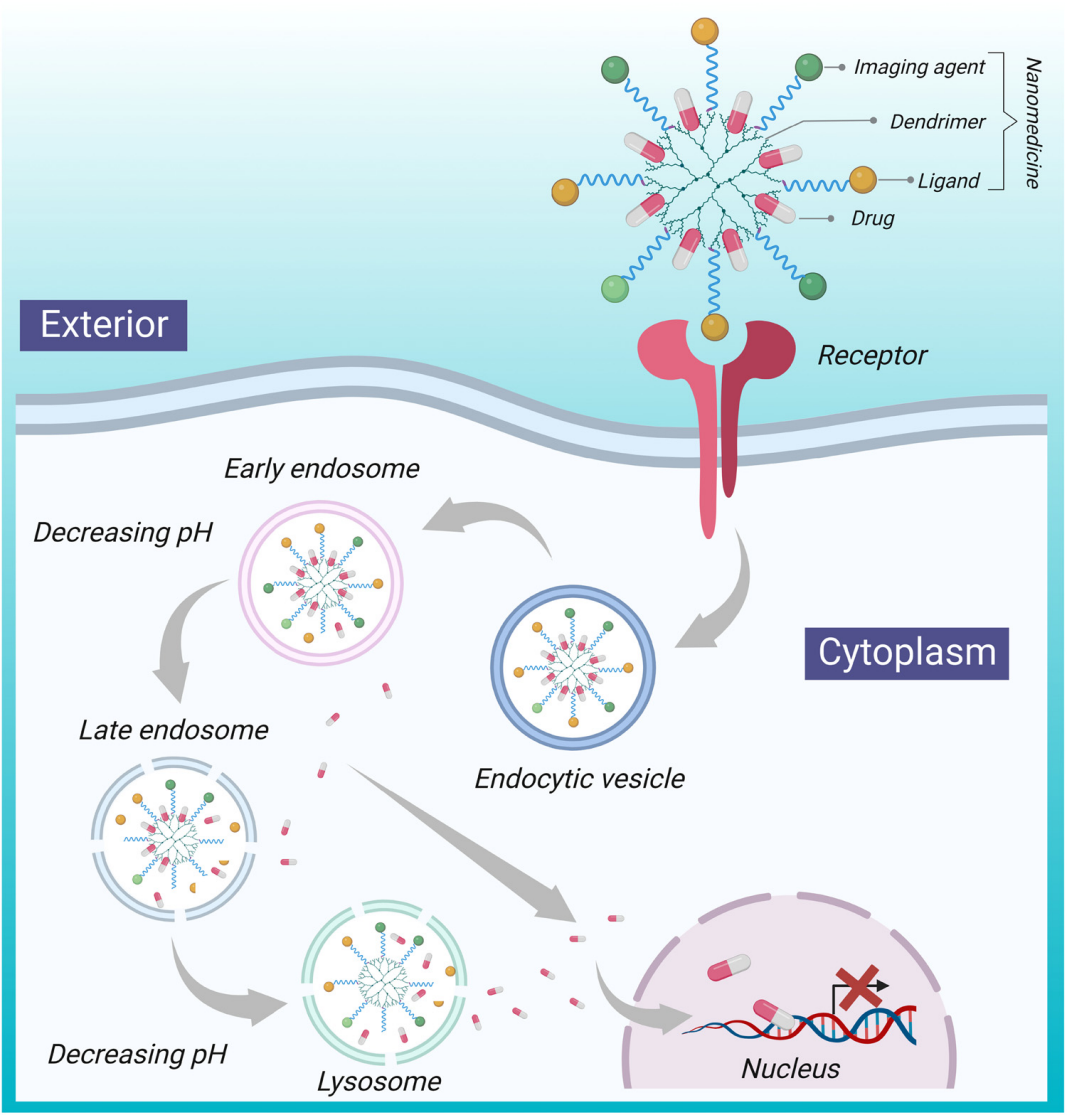
Nanoparticle (like doxorubicin and methotrexate) mechanism [93] for drug delivery to the brain using dendrimers. Nanomedicine, which is difficult to pass through the BBB, could be transferred using dendrimer, with the presence of ligands that are recognized by receptors present on the exterior of the BBB.

Physiochemical properties of nanoparticles like small size, surface charge, hydrophilicity, and nanocarrier ligand targeting are some of the reasons why they are able to pass through the brain endothelial tissue. Because of the presence of electrostatic interactions between the carrier and cells, the positive charge on the surface of the nanoparticle and the negative charge on the endothelial cells of the brain interact. The lipophilic nature of the nanomaterials enhances their properties and accelerates the adsorption process, as it associates with the solubility and permeability of the compound [85,86,87]. The nanoparticle gets absorbed via endocytosis and transcytosis on the brain endothelial cells containing low-density lipoprotein receptors, followed by desorption [88]. Due to this desorption, the particles return to the bloodstream, and they release the drug encapsulated inside them on BBB’s surface, which diffuses through the parenchyma of the brain. Human blood capillaries have an average diameter of 6–9 μm and the cell diameter is 20 μm, which allows nanoparticles to easily pass through the blood capillary endothelial cells due to their small size, via transport mechanisms like endocytosis and transcytosis [89]. These drug-carrying nanoparticles are endocytosed, and they enter further inside the cell or the parenchyma brain. Although the drug delivery mechanism of nanoparticles has not been fully explored, they are believed to work by several mechanisms [90,91,92]. One suggested mechanism is the solubilization of the endothelial cell membrane lipids by the effect of general surfactant, allowing fluidization of membrane and increasing permeability across the BBB. Another suggested mechanism is the inhibition of the efflux system like P-gp. P-gp is a protein that is expressed in endothelial cell plasmatic membranes of the BBB, and it protects the brain by not allowing the passage of substances that could cause damage to the cells. Nanoparticles could also act by inducing brain vasculature toxicity. Opening the TJs is another way in which nanoparticles pass through the BBB. Processes like transcytosis and endocytosis through the layer of endothelial cells are facilitated by nanoparticles’ increased absorption in the walls of the capillary and retention in the brain–blood capillaries. This results in an increased concentration gradient, which theoretically leads to an increase in transportation through the layers of endothelial cells. However, it is not a good mechanism to deliver drugs across the BBB because the drug that is being diffused can efflux out due to several transporters like P-gp. A drug delivery mechanism of dendrimers is shown in Figure 1.

## 5 Nanotechnology application in the treatment of various NDDs

Nanotechnology provides a new solution to problems associated with CNS disorders. Various CNS disorders, such as multiple sclerosis (MS), PD, ALS, Huntington’s disease (HD), AD, and schizophrenia that are almost incurable and have a few treatment options, could be cured with the help of several nanoparticles (Table 1).

**Table 1:** Summary of application of NPs in the treatment of CNS disorders

Disease	Nanocarrier used	Drug loaded	Animal models/cell line used	Outcomes/effects/treatment	References
AD	Polysorbate 80-coated poly(*n*-butyl cyanoacrylate) nanoparticle	Tacrine	Mice	Large amount of tacrine was found in the brain	[95]
	Poly(*n*-butyl cyanoacrylate) nanoparticles coated with polysorbate 80	Rivastigmine	Mice	A large amount of rivastigmine was able to pass through the BBB	[96]
	CPP (modified liposomes)	Rivastigmine	Murine BMVEC model	Rivastigmine liposomes especially CPP-Lp improve the brain delivery and enhance pharmacodynamics	[97]
	Fe_2_O_3_@CDs	Curcumin	PC12 cell lines	CUR-Fe_3_O_4_@CDs have a strong affinity toward Aβ and thus inhibit extracellular Aβ fibrillation. It also inhibits the Aβ-mediated production of ROS and neurotoxicity	[98]
	Chitosan nanoparticles (CS-NPs)	Sitagliptin	—	The use of SIT-CS-NPs was observed to increase the SIT levels in the brain by 5.07-fold in comparison with free SIT after administration	[99]
	Nanostructured lipid carrier	Transferrin functionalized nanocarriers	Endothelial cells of the brain	Encouraged expression of BCRP and P-gp, thus helping in clearance of amyloid β peptide on brain endothelial cells	[100]
PD	SLNs	Rasagiline	—	There were no changes in particulate matter, colloidal stability, and drug encapsulation effectiveness	[110]
	Manganese oxide-based nanoparticles	Levodopa	Pig and rat	The analysis indicates that perhaps the nanoparticle production of Mn^2+^ induced a time-dependent alteration	[112]
	Chitosan-coated nanoparticles	Levodopa liposomes	Rats	Endeavor to lessen the harmful effects of levodopa	[113]
	Chitosan-coated nanoparticles	Selegiline (Emsam) and rotigotine	Wistar rats	R-HCl transport toward the brain can indeed be strengthened, which could also help with therapeutics	[114]
	PHBV as well as T80-coated chitosan	Pramipexole	Mice	Substances that have recently been developed to reduce motor twitches for an instance safinamide as well as opicapone	[115]
	Graphene oxide	Trihexyphenidyl hydrochloride	—	Trihexyphenidyl hydrochloride showed sustained release behavior with release efficiency of 89–98%	[116]
	CNM-Au8	—	—	Protects motor neurons from death and severe damage in preclinical experiments	[118]
HD	Nanoparticles loaded with siRNA	—	—	Nanocarriers loaded with siRNA were administered from the nose directly to the brain to target the striatum and cerebral cortex	[121]
MS	Fluorescent phosphorhydrazone dendrimers	—	HeLa (human epithelioid cervical carcinoma), HUVEC (human umbilical vein endothelial) cells, and HEK 293 (human transformed primary embryonal kidney) cell lines	The dendrimer worked as an anti-inflammatory agent and was suggested for treating MS	[132]
	LiF-Nano-formulations with PLGA	—	CD4	LiF-Nano-CD4 was successfully able to pass through BBB and also exhibited anti-inflammatory effects in frontal cortex	[133]
ALS	Carbon graphene nanoparticle	—	—	Graphene-based nanoparticles may help in delivering miRNA for the treatment of ALS	[136]

### 5.1 AD

AD is identified by memory and cognitive dysfunction. It involves the accumulation and uneven folding of amyloid plaques (Amyloid β, Aβ) in the hippocampus, and these plaques lead to the formation of senile plaques (SPs) and neurofibrillary tangles (NFTs) from the buildup of tau protein. In the United States, this disease is the sixth leading cause of disease-related deaths and fifth among disease-related deaths in the aged group around 65 years old. AD is characterized by the presence of Aβ_42_ fibrils; the imbalance in the synthesis and clearance of Aβ in the brain causes neurotoxicity and immune-inflammatory response, resulting in AD. Hence, some of the many ways to treat AD involve the removal of Aβ deposition, reduction of Aβ deposition, or protection of the neurons in the brain; however, there is no complete treatment or therapy yet available for this disease [94]. Although there are numerous problems associated with the currently present therapies of AD, intranasal techniques are the only solution, which appears to be working. The drugs currently present for treating AD are neurotransmitter- or enzyme modulation-based. These drugs have adverse effects on the patients, which results in the patients discontinuing treatment. For example, acetylcholinesterase inhibitors show gastrointestinal side effects like vomiting and nausea. Tacrine needs to be administered per day because of its short life span. Similarly, rivastigmine and galantamine need to be administered twice a day. Another drug memantine, which helps in the management of AD, could lead to problems like vomiting, constipation, confusion, and dizziness. Nanotechnology provides a new solution for the diagnosis and treatment of AD. Since the only problem is the inability of the drug to reach the target site, the drugs could be transferred to the brain with the aid of nanoparticles. Many nanoparticles have been found to be suitable for treating AD, like liposomes, liquid crystals, solid lipid carriers, microemulsions, hydrogels, and PNPs and SLNs. In a study, tacrine was loaded in polysorbate 80-coated poly(*n*-butyl cyanoacrylate) nanoparticles that were developed by emulsion polymerization. Tacrine concentration was not sufficient in the lungs and kidneys, so the author suggested delivering drug coated in polysorbate 80 nanoparticles to the brain through the interaction of the endothelial cells of the brain and polysorbate 80 coatings [95]. Mice were injected with the nanoparticle to study the effects. When coated nanoparticles were used, the amount of tacrine was significantly increased compared to when non-coated nanoparticle administration or the drug alone was used. In another similar study, the author used polysorbate 80 covering to construct poly(n-butyl cyanoacrylate) nanoparticles to deliver rivastigmine to treat AD [96]. To enhance the reach of rivastigmine to the brain, a scientist prepared a CPP that altered liposomes and rivastigmine liposomes, minimizing the side effects and improving the pharmacodynamics. A higher concentration of rivastigmine was able to pass the BBB compared to when only the drug without the nanoparticles was delivered [97]. In a recent study, curcumin was used for the therapy of AD via Fe_2_O_3_@CDs. Rat pheochromocytoma (PC12) cell lines were used to form Fe_3_O_4_@CDs nanocomposite, and CDs were loaded with curcumin by hydrogen-bonding interaction and π–π accumulation. Aβ fibril decomposition was observed [98]. Furthermore, when nanoparticles loaded with sitagliptin (SIT), a dipeptidyl peptidase-4, were administered, significant therapeutic effects against AD symptoms were observed [99]. In a recently performed study, a new approach was found for AD therapy. It was to induce breast cancer resistance protein transporters and P-gp expression by using MC_11_ on the endothelial cells of the brain [100]. The nanostructured lipid carrier that is transferrin functionalized is able to induce the expression of a protein that could possibly be helpful in AD therapy. These observations indicate that nanoparticles could have a significant role in the AD treatment and possible curative effects.

### 5.2 PD

This is a progressive neurodegenerative ailment that restricts movement of affected individuals. Increased oxidative stress, amplification of alpha-synuclein, and exposure to 1-methyl-4-phenyl-1,2,3,6-tetrahydropyridine are only a few of the many sources that cause neuron degradation of the substantia nigra, which leads to induction of PD. PD results in the death or disintegration of certain nerve cells (neurons) in the brain [101]. PD is random or familial degeneration of brain’s nerve cells that limit their capacity to generate the neuronal transmitter dopamine [102]. The loss of neurons that produce dopamine, a chemical messenger in the brain, is responsible for some of the symptoms. When dopamine levels fall, abnormal brain activity ensues, leading to decreased movement and other symptoms. Symptoms may progress quickly, beginning with a nearly imperceptible trembling of one arm. Tremors are quite prevalent. The condition can also produce stiffness or sluggishness in movement. Despite being the second most common NDD, PD lacks definite therapeutic options and successful medication delivery.

Several techniques are available to generate materials alone, and their intended application or function varies according to the type of material chosen. For example, inorganic materials are frequently used to create nanosystem metals or QDs, which are then used to build delivery-imaging agents [103]. Using a composition consisting of hydrogel and a microporous polyethylene membrane, Fang et al. created a unique transdermal device to distribute selegiline (Solupor). Even with hydrogel in the formulation, the outcomes demonstrated that perhaps the Solupor barrier was the rate-limiting factor [104]. The new transdermal device devised by Azeem et al. might assure continuous ropinirole administration and avoid the initiation of levodopa motor problems. The end evaluation showed a 7.5-fold improvement in ropinirole skin penetration in *in vitro* experiments [105]. Nanoparticles have been shown to enable efficient medication administration, enhance the bioavailability, reduce pharmacokinetic adverse effects, reduce drug dose, and increase the target delivery against a number of ailments directed at the brain [106]. Lipid-based nanoparticles have been shown to be biocompatible. The rasagiline-loaded SLNs were formulated using an analytic hierarchy technique, a microemulsion method that resulted in an 83.6% yield [107]. Organic materials too have been associated with improved biocompatibility and lower material toxicity [108]. Malfunctions of the basal ganglia, which can cause movement problems, are a few of the incidents that set off the timeline of reasoning that can lead to PD [109]. The *in vitro* release of rasagiline from SLNs was investigated in another study. The results revealed a biphasic controlled release with a preliminary 20% release over 30 min, followed by an analysis from the *in vitro* release drug study of rasagiline-enhanced SLNs in the same group [110]. For example, rasagiline-loaded SLNs were sterilized using moist heat, and the findings showed no alterations in the size of particles, zeta potential, or drug encapsulation efficiency [111]. The scientists also created nanoparticles using manganese oxide. In another work, they were synthesized and characterized with levodopa for application as a revolutionary MRI distinction agent-combined drug delivery system. The analysis showed that the emission of Mn^2+^ from the nanoparticles caused a time-based change in MRI contrasts from dark to brilliant [112]. Cao et al. employed chitosan-coated levodopa liposomes to study the responses and impacts of various compounds in an attempt to reduce levodopa’s negative effects [113]. Currently, transdermal patches are used to alleviate PD, while selegiline (Emsam) and rotigotine (Neupro) are marketed for clinical depression. Increased dopamine levels, mimicked dopamine action, and low dopamine metabolization, as well as storage, are currently the major strategies for treating motor symptoms. Ray et al. created chitosan nanoparticles to effectively encapsulate ropinirole and allow its passage through the BBB. When comparing non-coated chitosan nanoparticles to T80-coated chitosan nanoparticles, the results showed that the T80 coating resulted in increased brain accumulation [114]. Javan and Hasab developed a lengthy formulation with pramipexole incorporated in polymeric poly(3-hydroxybutyrate-*co*-3 hydroxyvalerate) (PHBV)-based nanoparticles [115]. Jawanjal et al. created a graphene oxide to administer trihexyphenidyl [116]. The current pharmacological treatment focuses on symptomatic alleviation of both motor and non-motor complaints [117]. In preclinical investigations, oral suspension of gold nanocrystals, CNM-Au8, could perhaps endorse bioenergetic cellular activities as well as assist in removing harmful toxins of cell metabolism, which may cause motor neuron collapse in patients with ALS or PD [118]. They have also been demonstrated to safeguard motor neurons against apoptosis as well as major trauma and are currently in Phase 2 clinical trials [118].

### 5.3 HD

This is a specific type of neurological disorder induced by increased CAG trinucleotide repetition in the gene responsible for producing a protein called huntingtin (HTT), which is essential for conventional development before birth. Accumulation of HTT mutants decreases the histone acetylation, and this decrease is associated with neural damage and loss in HD. HD is incurable and leads to cell death in the brain, further causing motor, cognitive, and psychiatric disorders [119]. There is no therapy known which resists or halts the progression of HD. Mitochondria have a significant role in HD pathogenesis as it controls the energy metabolism and apoptosis pathways, and defected mitochondria and oxidative stress can be readily detected in biological material collected from the patient [120].

Recently, a clinical trial studied the acceptability, security, and properties of the Ionis-HTTRx in adults with early HD [120]. An anti-sense oligonucleotide was designed to hinder HTT mRNA and thereby decrease the concentration of mutant HTT. However, there was an obstacle: the application of this treatment is related to efficient and speedy drug supply method, and thus, in reality, it will be required to be administered at least six times per year throughout patient lifetime. In the diagnostic procedure, a lumbar puncture is performed but because of the chronic portal to the brain, there are chances of unfortunate side effects such as hemorrhage, local infection, and radiculopathy. To overcome this obstacle, many laboratories are working on developing a series of nanocarriers loaded with siRNA to distribute it from the nose directly to the brain to target the striatum and cerebral cortex [121].

There was one more trial studying the same factors, and it concluded that after treatment with selenium nanoparticles, HTT aggregation and *in vivo* reactive oxygen species levels in transgenic *Caenorhabditis elegans* Huntington model were reduced and HDA mRNA was downregulated compared to epidemic levels as Nano-Se acts as an antioxidant to regulate the expression of the hda family. Nano-Se less than 2 μM could relieve the behavioral dysfunction, overcome neuronal death, and can offer safety to *C. elegans* under stress [122]. Therefore, Nano-Se can possibly play an essential and promising role in treating HD in the coming future.

### 5.4 MS

MS is identified by the demyelination of the neurons. Demyelination is a process in which myelin, present as a protective coating on the neurons, is damaged. MS is an autoimmune disease and occurs when the T and B cells attack the nervous system, causing demyelination [123]. This disease is found more in younger individuals and is more prominent in females [124]. CNS inflammation and demyelinated neurons are indications of this disease [125].

The diagnosis of this disease is difficult due to its non-specificity. A variety of assessment and imaging techniques are present for diagnosis, but the agents lack the capability to pass through the BBB and lose their strength halfway during delivering [126]. These traditional methods of imaging and uncovering lesions depend upon the strength of the field, factors of imaging, and contrasting agent dosage [127,128,129]. Nanotechnology is a great alternative in the diagnosis and treatment of MS. The contrast agents for the diagnosis of cellular inflammation have been able to better reach to the target site due to their small size.

Nanoparticles are usually used in two ways: one in which they are used as a neuroprotectant and another in which they act as an anti-inflammatory agent, or both. Liposomes were found to be effective in treating MS. One of the main causes of MS is the generation of antibodies against myelin basic protein (MBP). Mannosylation of liposomes loaded with fragments of MBP could reduce the activity of anti-MBP. This could lead to the uptake of mannose by the receptors on the macrophages [130]. The ability of liposomes to reach the targeted site could be increased by surface conjugation with glucose, transferrin, specific peptides, lactoferrin, etc. Surface conjugation provides liposomes with the ability to cross the BBB and deliver the drug to the target site. Dendrimers are also effective against MS. Their ability to act on microglial cells and astrocytes that are involved in inflammation makes them suitable for the treatment of MS [131]. A study suggested that fluorescent phosphorhydrazone dendrimers could work as anti-inflammatory agents and could be effective for treating MS [132]. Other commonly used nanoparticles for the detection of inflammation in MS are gadolinium-DTPA (Gd-DTPA), superparamagnetic iron oxide nanoparticles (SPIONSs), and QDs. The nano-formulation of LIF (LIF-Nano) with poly(lactic-*co*-glycolic acid) (PLGA) is known to target CD4 cells with 1,000-fold increased potency. In a recent study, this LIF-Nano-CD4 was used with the longer-term aim of treating inflammatory lesions of MS. It was further observed to cross BBB and cargo-specific anti-inflammatory responses in frontal cortex of the brain [133].

### 5.5 ALS

ALS is also called motor neuron disease. It is a neurological disease that triggers muscular weakness and ultimately paralysis by affecting the lower and upper motor neuron. Its etiology is complex, involving genetic, cellular, and molecular pathways [134].

Depending on the family history, ALS may be classified as familial (FALS) or sporadic (SALS). Genetic mutations induce abnormalities in typical protein functions. Over 20 genes have been linked to ALS, with mutations or variations in fus, tardbp, c9orf72, pfn1, or Sod1 accounting for about 60–70% of FALS cases and 10% of SALS cases [135]. To date, there is no efficient treatment for ALS as there are numerous challenges like crossing the biological barriers such as BBB and blood–spinal cord barrier, as well as the significant innate features of the drugs themselves. However, nanotechnology-based strategies include nanoparticles that show great capacity to supply therapeutic operatives to overcome biological barriers, improve drug bio-stability, and improve interaction with target sites.

According to the studies on ALS, miRNAs deregulate the pathways involved, and due to this reason, the interventions were designed and developed to address miRNA expression anomalies [136]. To overcome the limitation of biological barriers, numerous viral and non-viral vectors have been constructed, and although the transfection efficiency is high with the viral system in comparison with the non-viral system, the non-viral system is preferred as it has a little or no size constraints on nucleic acids and there are fewer hazardous side effects. Non-viral substances are mostly extracellular vesicles and polymeric, inorganic, and lipid-based vectors [137].

Nanoparticles represent the most widely used non-viral transporters for transferring exogenous nucleic acids. To treat ALS, the most suitable option is two-dimensional graphene. Furthermore, when the nanoparticles, such as carbon graphene nanoparticles, are constructed in a smaller size than the usual, they are able to penetrate biological barriers such as the BBB [136].

### 5.6 Schizophrenia

Schizophrenia is characterized by psychological symptoms that make the patient decipher reality abnormally. It is a distinctive disordering of the patient’s course of thought of being controlled by outside forces, delusions, and hallucinations. Few early symptoms for the detection of the disease include insomnia or oversleeping, social withdrawal, depression, and bizarre way of speaking. Patients with schizophrenia are generally disabled and require lifelong treatment. Studies on its etiology and associated morbidity are still on going.

A recent study showed that sulpiride, a substituted benzamide anti-psychotic drug that has particular dopaminergic blocking activity, could be effective in treating schizophrenia. The main objective of the study was to describe the sulpiride molecular profile and interpret the sulpiride receptor. Another objective was to create a PNP with a carrier chitosan having benefits such as controlled drug release, to enhance the solubility and stability of the drug, improve the utility, and decrease toxicity [138].

## 6 Limitations and future directions

Recently, tailored medicines as well as interesting new approaches toward treating CNS illnesses have emerged with nanomedicine. It must be regarded as an effective method for delivering medications via the BBB [139,140]. Carriers may be engineered to address certain cells through signaling systems, diffuse across brain tissue, and breach the BBB while transporting therapeutic drugs. Because they have a greater surface area, which increases drug accessibility; are organic (natural or synthesized polymers) and otherwise inorganic (e.g., metals for nanophotonic prescription meds); may possess an ionic surface charge, which encourages favorable interactions for drug action and other functional areas (e.g., PEGylation); and also have precision for the objective via complex formation, nanomedicines have had the potential to possess an efficient administration of drugs framework (e.g., antibody labeling) [141,142]. The large majority of nanomedicine drugs being used to treat CNS illnesses has consistently explored as well as emphasized nanotechnology-based pharmaceutical delivery methods. Additionally, many nanodrugs currently undergoing research trials; nevertheless, it is currently unknown how they will be transported as well as how secure they will be [143,144]. Both qualities and makeup of nanoparticles may result in oxidative stress, instability inside the BBB [10,145], and a decomposition of amino acids, which would enhance brain neurotoxicity. Although functionalized nanoparticles offer effective therapeutic localization, their enormous surface area plus compact shape may cause particle aggregation, thus restrict drug loading. Increasing neuroinflammation, leading to oxidative stress, damage to DNA, and allergic responses could all be caused by the medication delivery process and also the dosage of nanomedicine used [146]. Consequently, knowledge of nanomedicine’s biodegradability and biocompatibility is necessary. In order to supply full evidence on the toxicity of nanoformulations in humans, it is indeed urgently necessary to devise and execute standardized tests to evaluate the toxicity of nanoformulations in both *in vitro* and *in vivo* studies.

Nanomedicine needs to engage systemically throughout neurons in order to be successful. Drug delivery might face more difficulties due to the neuronal level’s complex cellular connections and restricted anatomical accessibility. Maintaining the CNS’s critical functions as they were before to medication delivery is a significant problem.

Research findings of a variety of multifunctional nanomaterials with both the detailed controllable characteristics needed for nanotherapeutics have demonstrated extraordinary obstacles that require to be promptly resolved; for instance, a few of the micro have characteristics such as biological reasonableness, effectiveness, biocompatibility, and toxic effects in the living organism classifications [147]. Modifying as well as controlling nanotechnology, pharmaceutical cargo packing, medication transport toward the brain, deep brain stimulation (DBS), implantation stimulation, and even brain cell functioning are among the numerous difficult problems being worked upon [148]. In order to avoid unintended toxicity to healthy cells, the characterization as well as production of nanomaterials seems to be of utmost relevance for their implementation in medication administration. These developments should make it easier to develop treatments in the future depending on how severe a patient’s neurodegeneration becomes.

A limited handful of nanoformulations are also currently being evaluated, such as organic or inorganic nanocarriers through preclinical studies again for the reduction of cancer formation and enhanced lifespan [147,149]. A German firm called Magforce pioneered the NanoTherm® therapeutic that also uses ferrofluid consisting of SPIONSs. These nanoparticles have been claimed to have the capability to kill malignant cells through magnetic hyperthermia as well as radiation [150]. Some other indications are neural stimulation driven by magnetic-electro nanoparticles, which could be a significant implantable DBS therapy of the future. To encompass tissue and cellular the brain’s functions in numerous places, this nanosystem can be designed to create magnetothermic, magnetoelectric, acoustoelectric transformations, photothermal, and photoelectric. In accordance with the needs of the cellular response or brain tissue, the management of such stimulating and excitatory qualities may be tuned [151].

## 7 Conclusion

Due to the constant deterioration of the lifestyle and environment, the frequency of people with CNS disorders like PD, AD, MS, ALS, and brain tumors is constantly rising. The main challenge of treating these disorders is the impermeability of the BBB and the blood–cerebrospinal fluid barrier, due to their unique environment, sensitivity toward foreign materials, and complex structure, which makes it difficult to deliver drugs through them. There is a need for the development of a strong and effective carrier that could deliver the drugs efficiently to the target site with minimal or no side effects.

Nanoparticles have been proven to be effective and strong carriers in comparison with the conventional therapy for addressing CNS disorders. Nanoparticles, such as liposomes, nanotubes, nanopharmaceuticals, micelles, and nanosensors, are in high demand as theranostics for neuroprotection. Currently, nano-based approaches for neuroprotection and regeneration are sought after to address neurological cells’ physiology, biology, and pathology. Different kinds of nanoscalic approaches such as the reduction of reactive oxygen species and Aβ oligomerization have been proven to be effective for treating neurological disorders. Nanoformulations of numerous drugs like edaravone, curcumin, and nerve growth factors are generating great interest for neurological diseases; however, there are little data available on their adverse effects. Nanotechnology has a promising future in CNS disorder treatment due to the ability of nanoparticles to pass through the BBB, flexibility to be engineered according to the need, and targeted delivery. Nanotechnology could easily aid us to enhance our approaches for neurological disorder treatment.

Nonetheless, nanotechnology has conclusively demonstrated to be an innovative and encouraging scientific knowledge source that enables precise and easy delivery of drugs to the CNS. However, we have more to learn regarding the characteristics and traits of nanoparticles to analyze their dynamic characteristics in biomedical science [152]. At the moment, there is no multidimensional prescription medication for various neurological diseases caused by dysregulation of multiple independent biochemical pathways [153]. Nanodrugs have the potential to address this limitation. Multiple gene intervention in neuronal cells has been recognized as a challenging objective, and nanotechnology-based delivery of drugs could presumably be an efficient method to achieve this in CNS therapies.

The growing population, as well as the prevalence of brain disorders, necessitates the urgent research of new therapeutic approaches with good potential. Applications of nanotechnology throughout neuroscience would be able to address unmet clinical requirements and provide patients with positive outcomes. A new generation of nanomedicine could be capable of controlling long-lasting drug release as well as the delivery of drugs to a specific target. Rather than reducing adverse effects and increasing the survivability of nanodrugs, efforts must be made toward improving nanotechnological methodologies for all pharmaceutical drugs to increase understanding of the correct target site and subsequently improve living standards. We cannot reject the perceived impact of nanomedicines, but their own prospect and hazardous procedures point to possible negative consequences. Given the technological advance of nanotechnology through contemporary research, one cannot immediately disregard it based on its drawbacks. To eliminate the adverse effects of nanotechnology, specialized guidelines must be followed. It can be anticipated that the nanotechnology-based delivery system might revolutionize traditional pharmaceutical delivery and the generated modified pharmaceuticals will be significantly more productive than the currently accepted method.
